# Supplementary material for: Intermittent rate coding and cue-specific ensembles support working memory
Source: Nature. 2024 Nov 6;636(8042):422–9. doi: 10.1038/s41586-024-08139-9 (PMC11634780; doi:10.1038/s41586-024-08139-9)
Supplement: Supplementary file 1 — Reporting Summary [file 41586_2024_8139_MOESM1_ESM.pdf]

nature portfolio

Corresponding author(s): Matthew Panichello
Tirin Moore

Last updated by author(s): Nov 20, 2023

Reporting Summary

Nature Portfolio wishes to improve the reproducibility of the work that we publish. This form provides structure for consistency and transparency in reporting. For further information on Nature Portfolio policies, see our Editorial Policies and the Editorial Policy Checklist.

Statistics

For all statistical analyses, confirm that the following items are present in the figure legend, table legend, main text, or Methods section.

n/a	Confirmed	
☐	☒	The exact sample size (n) for each experimental group/condition, given as a discrete number and unit of measurement
☐	☒	A statement on whether measurements were taken from distinct samples or whether the same sample was measured repeatedly
☐	☒	The statistical test(s) used AND whether they are one- or two-sided<br>*Only common tests should be described solely by name; describe more complex techniques in the Methods section.*
☒	☐	A description of all covariates tested
☐	☒	A description of any assumptions or corrections, such as tests of normality and adjustment for multiple comparisons
☐	☒	A full description of the statistical parameters including central tendency (e.g. means) or other basic estimates (e.g. regression coefficient) AND variation (e.g. standard deviation) or associated estimates of uncertainty (e.g. confidence intervals)
☐	☒	For null hypothesis testing, the test statistic (e.g. F, t, r) with confidence intervals, effect sizes, degrees of freedom and P value noted<br>*Give P values as exact values whenever suitable.*
☒	☐	For Bayesian analysis, information on the choice of priors and Markov chain Monte Carlo settings
☒	☐	For hierarchical and complex designs, identification of the appropriate level for tests and full reporting of outcomes
☒	☐	Estimates of effect sizes (e.g. Cohen's d, Pearson's r), indicating how they were calculated

Our web collection on statistics for biologists contains articles on many of the points above.

Software and code

Policy information about availability of computer code

Data collection	Stimuli were presented and behavioral responses registered using Psychtoolbox (version 3) and Matlab R2022a. Code for stimulus presentation is available at https://github.com/panichem/SingleTrialDynamics. Neuropixels data were acquired using SpikeGLX. Waveforms were sorted using Kilosort3.
Data analysis	Data were analyzed using built-in functions and custom code written in Matlab. Built-in functions are noted in the associated methods section (e.g., fitclinear for logistic regression). Equations for non-standard statistics are provided in the methods.<br>Code for custom functions are provided in the manuscript as a reference to the original source or are available on GitHub (https://github.com/panichem/SingleTrialDynamics).

For manuscripts utilizing custom algorithms or software that are central to the research but not yet described in published literature, software must be made available to editors and reviewers. We strongly encourage code deposition in a community repository (e.g. GitHub). See the Nature Portfolio guidelines for submitting code & software for further information.

Data

Policy information about availability of data

All manuscripts must include a data availability statement. This statement should provide the following information, where applicable:

- Accession codes, unique identifiers, or web links for publicly available datasets
- A description of any restrictions on data availability
- For clinical datasets or third party data, please ensure that the statement adheres to our policy

The data underlying this study are available on Dryad (https://doi.org/10.5061/dryad.kkwh70sct).

Research involving human participants, their data, or biological material

Policy information about studies with human participants or human data. See also policy information about sex, gender (identity/presentation), and sexual orientation and race, ethnicity and racism.

Reporting on sex and gender	n/a
Reporting on race, ethnicity, or other socially relevant groupings	n/a
Population characteristics	n/a
Recruitment	n/a
Ethics oversight	n/a

Note that full information on the approval of the study protocol must also be provided in the manuscript.

Field-specific reporting

Please select the one below that is the best fit for your research. If you are not sure, read the appropriate sections before making your selection.

☒ Life sciences ☐ Behavioural & social sciences ☐ Ecological, evolutionary & environmental sciences

For a reference copy of the document with all sections, see nature.com/documents/nr-reporting-summary-flat.pdf

Life sciences study design

All studies must disclose on these points even when the disclosure is negative.

Sample size	As detailed in the manuscript, a total of 8,225 single and multi-unit neurons were recorded from 3 animal subjects across 25 experimental sessions. The number of subjects (3) is above the standard in the field (Fries & Maris, 2022). The number of neurons is in line with the current state-of-the-art (Trautmann et al., 2023).
Data exclusions	Neurons with very low firing rates were excluded from further analysis. Exclusion criteria are detailed in the methods.
Replication	Independent experiments were performed in 3 animals. All data were analyzed, except for the exclusion criteria described above. There were no failed replication attempts (i.e., no animals failed to learn the task and no neural recordings were excluded).
Randomization	Each animal was exposed to every task manipulation. Within a session, task manipulations were randomized across trials. Neurons were recorded without bias, with electrodes placed to maximize signal-to-noise of the electrophysiological signal.
Blinding	All animals were assigned to a single experimental group, and so blinding was not necessary or possible. However, experimenters were blinded to experimental conditions during recording of neurons and during sorting of waveforms into single neurons.

Reporting for specific materials, systems and methods

We require information from authors about some types of materials, experimental systems and methods used in many studies. Here, indicate whether each material, system or method listed is relevant to your study. If you are not sure if a list item applies to your research, read the appropriate section before selecting a response.

Materials & experimental systems

n/a	Involved in the study
☒	☐ Antibodies
☒	☐ Eukaryotic cell lines
☒	☐ Palaeontology and archaeology
☐	☒ Animals and other organisms
☒	☐ Clinical data
☒	☐ Dual use research of concern
☒	☐ Plants

Methods

n/a	Involved in the study
☒	☐ ChIP-seq
☒	☐ Flow cytometry
☒	☐ MRI-based neuroimaging

Animals and other research organisms

Policy information about studies involving animals; ARRIVE guidelines recommended for reporting animal research, and Sex and Gender in Research

Laboratory animals	Subjects were three adult male rhesus macaques (Macaca mulatta), ages 8, 11, and 12.
Wild animals	No wild animals were used in the study
Reporting on sex	As monkey electrophysiology studies typically report findings from only a few subjects (Fries & Maris, 2022), isolating effects due to gender is typically not possible. All research subjects in the present study are male.
Field-collected samples	No field-collected samples were used in this study.
Ethics oversight	All experimental procedures were approved by the Stanford University Animal Care and Use Committee and were in accordance with the policies and procedures of the National Institutes of Health.

Note that full information on the approval of the study protocol must also be provided in the manuscript.

Plants

Seed stocks	n/a
Novel plant genotypes	n/a
Authentication	n/a
